#  Judicialização de produtos à base de canabidiol no Brasil: uma análise de 2019 a 2022 

**DOI:** 10.1590/0102-311XPT024723

**Published:** 2023-10-09

**Authors:** Ronaldo Portela, Daniel Marques Mota, Paulo José Gonçalves Ferreira, Mariana Dias Lula, Bruno Barcala Reis, Helian Nunes de Oliveira, Cristina Mariano Ruas

**Affiliations:** 1 Faculdade de Farmácia, Universidade Federal de Minas Gerais, Belo Horizonte, Brasil.; 2 Agência Nacional de Vigilância Sanitária, Brasília, Brasil.; 3 Defensoria Pública do Estado de Minas Gerais, Belo Horizonte, Brasil.; 4 Faculdade de Medicina, Universidade Federal de Minas Gerais, Belo Horizonte, Brasil.

**Keywords:** Canabidiol, Cannabis, Judicialização da Saúde, Avaliação de Tecnologias em Saúde, Cannabidiol, Cannabis, Health’s Judicialization, Biomedical Technology Assessment, Cannabidiol, Cannabis, Judicialización de la Salud, Evaluación de la Tecnología Biomédica

## Abstract

Este estudo analisou as ações judiciais de pacientes que solicitaram ao Sistema Único de Saúde produtos à base de canabidiol (CBD) durante o período de 2019 a 2022, descrevendo características sociodemográficas, clínicas e jurídicas. Trata-se de um estudo transversal composto pela avaliação das notas técnicas emitidas pelos Núcleos de Apoio Técnico do Judiciário (NatJus), que embasaram as decisões judiciais. Os dados foram obtidos do sistema e-NatJus, do Ministério da Justiça, utilizando técnicas de *web scraping*. Regressão logística foi empregada para estimar razões de chances com intervalos de 95% de confiança. Foram analisadas 1.115 notas técnicas das ações demandantes de CBD, das quais 54,7% dos pacientes eram do sexo masculino, com idade média de 18,4 anos, em sua maioria da Região Sul do país (38,8%), e 49,6% buscavam tratamento para epilepsia. Das ações com pareceres favoráveis, 28,8% não tinham evidências científicas, 26,5% pleitearam produtos sem registro na Agência Nacional de Vigilância Sanitária e 25,3% dos que tinham registro não estavam em conformidade com a indicação terapêutica. Os pacientes da Região Nordeste tiveram a chance de parecer favorável aumentada em 3 vezes; e os que tinham diagnóstico de epilepsia, em 2,3 vezes. Os pareceres técnicos que deram suporte aos magistrados para as decisões judiciais das demandas de pacientes por produtos à base de canabidiol no Brasil estavam, em sua maioria, em conformidade com evidências científicas, denotando a importância dos NatJus na qualificação do acesso a produtos medicinais no país.

## Abstract (en)

The study analyzed the lawsuits of patients who requested cannabidiol (CBD)-based products from the Brazilian Unified National Health System during the period from 2019 to 2022, describing sociodemographic, clinical, and legal characteristics. This is a cross-sectional study composed of the evaluation of the technical notes issued by the Center for Technical Support of the Judiciary (NatJus), which supports judicial decisions. The data were obtained from the e-NatJus system, of the Brazilian Ministry of Justice, using web scraping techniques. Logistic regression was used to estimate odds ratios with 95% confidence intervals. We analyzed 1,115 technical notes of the CBD plaintiffs, of which 54.7% of the male patients, with a mean age of 18.4 years, mostly from the South Region of the country (38.8%), and 49.6% sought treatment for epilepsy. Regarding the actions with favorable opinions, 28.8% had no scientific evidence, 26.5% pleaded for products without registration with the Brazilian Health Regulatory Agency, and 25.3% of those that had registration were not in compliance with the therapeutic indication. Patients from the Northeast Region had a chance of a favorable opinion increased by 3.0 times and those diagnosed with epilepsy by 2.3. The expert opinions that supported the magistrates for the judicial decisions regarding the demands of patients for cannabidiol-based products in Brazil were mostly in accordance with scientific evidence, denoting the importance of NatJus in the qualification of access to medicinal products in the country.

## Abstract (es)

El estudio analizó las acciones legales de pacientes que solicitaron al Sistema Único de Salud brasileño productos a base de cannabidiol (CBD) durante el período de 2019 a 2022, describiendo características sociodemográficas, clínicas y legales. Se trata de un estudio transversal compuesto por la evaluación de las notas técnicas emitidas por los Núcleos de Apoyo Técnico del Poder Judicial (NatJus) que basaron las decisiones judiciales. Los datos se obtuvieron del sistema e-NatJus, del Ministerio de Justicia brasileño, mediante técnicas de web scraping. La regresión logística se empleó para estimar los *odds ratios* con intervalos del 95% de confianza. Fueron analizadas 1.115 notas técnicas de las acciones demandantes de CBD que tenían 54,7 % de los pacientes del género masculino, con una edad media de 18,4 años, en su mayoría de la Región Sur del país (38,8%) y 49,6% buscaban tratamiento para la epilepsia. De las acciones con opiniones favorables, el 28,8% no tenían evidencias científicas, el 26,5% pleitearon productos sin registro en la Agencia Nacional de Vigilancia Sanitaria y el 25,3% de los que tenían registro, no estaban en conformidad con la indicación terapéutica. Los pacientes de la Región Nordeste tuvieron la posibilidad de opiniones favorables aumentada en 3,0 veces y los que tenían diagnóstico de epilepsia en 2,3. Los dictámenes técnicos que dieron apoyo a los magistrados para las decisiones judiciales de las demandas de los pacientes por productos a base de cannabidiol en Brasil estaban en su mayoría en conformidad con las evidencias científicas, denotando la importancia de NatJus en la calificación del acceso a productos medicinales en el país.

## Introdução

O canabidiol (CBD) é um fitocanabinoide, que pode ser extraído da planta *Cannabis sativa* ou obtido sinteticamente. Os produtos à base de CBD têm sido utilizados para o tratamento de diferentes condições clínicas. Formulações de diferentes origens, concentrações e grau de pureza podem ser encontradas no mercado, muitas delas sem aprovação por órgãos reguladores, o que aumenta os riscos potenciais de ocorrência de eventos adversos à saúde humana ^1^.

As evidências científicas sobre a efetividade desses produtos são escassas. Entretanto, a garantia do acesso ao tratamento de enfermidades com CBD tem ocorrido especialmente pela via judicial, em alguns casos mesmo sem aprovação regulatória ^1^^,^^2^.

Devido aos seus efeitos terapêuticos promissores, o CBD, um composto terpenofenólico de 21 carbonos, é um dos canabinoides mais estudados atualmente. Esse composto não psicoativo, diferentemente do delta-9-tetraidrocanabinol (Δ9-THC), tem alegadas propriedades anti-inflamatórias, analgésicas, ansiolíticas e antitumorais. Além disso, existem relatos de uso para o tratamento de pacientes com transtorno de humor e epilepsia ^3^^,^^4^^,^^5^. Metanálises evidenciaram benefícios satisfatórios do uso do CBD na redução da frequência de convulsões, com toxicidade tolerável, em pacientes com epilepsia de difícil controle como a síndrome de Lennox-Gastaut, síndrome de Dravet e complexo de esclerose tuberosa ^6^^,^^7^^,^^8^^,^^9^.

Com exceção dos estudos que avaliam o tratamento da epilepsia, as demais pesquisas publicadas geralmente apresentam baixa qualidade metodológica e fraca evidência científica. Apesar disso, os produtos à base de CBD estão sendo utilizados no tratamento de várias condições clínicas ^1^^,^^2^. Ensaios clínicos que investiguem a eficácia para o tratamento da dor, doenças autoimunes, transtornos psiquiátricos e outras condições são necessários antes que o CBD possa ser recomendado para tratamento viável e seguro ^6^^,^^7^^,^^8^^,^^9^.

O uso de produtos à base de CBD é atualmente aprovado pela Agência de Alimentos e Medicamentos dos Estados Unidos (Food and Drug Administration - FDA), pela Agência de Saúde do Canadá (Health Canada) e pela Agência Europeia de Medicamentos (European Medicines Agency - EMA). A aprovação é específica para determinadas indicações terapêuticas, em alguns casos, enquanto em outros a escolha da indicação pode ser uma decisão médica. Apesar da aprovação, a falta de padronização e regulamentação levanta preocupações sobre a composição desses produtos ^2^^,^^10^.

No Brasil, em 2015, a Agência Nacional de Vigilância Sanitária (Anvisa) excluiu o CBD da lista de substâncias proibidas, incluindo-o na lista de substâncias sujeitas a controle especial por meio da *Resolução da Diretoria Colegiada* (RDC) *nº 3*, de 26 de janeiro de 2015 ^11^. Em seguida, a RDC nº 17, de 6 de maio de 2015 ^12^, definiu critérios e procedimentos para a importação por pessoa física de produtos à base de CBD associados a outros canabinoides para uso próprio, mediante prescrição médica. A RDC nº 327, de 9 de dezembro de 2019 ^13^, dispõe sobre os procedimentos e requisitos para a concessão de autorização sanitária para fabricação, importação, comercialização e fiscalização de produtos originados da *C. sativa*. Dez produtos à base de CBD e oito à base de extratos de *C. sativa* têm registro ativo na Anvisa ^12^^,^^14^.

As recentes alterações nas normas regulatórias no que tange aos produtos à base de CBD permitem maior acesso pelos pacientes, inclusive pelas vias judiciais. As demandas por esses produtos se juntam às demais, relacionadas aos bens e serviços de saúde que o Estado é obrigado a fornecer por ordem judicial. Esse fenômeno, conhecido como judicialização da saúde, tem gerado desafios e exigido dos Poderes Executivo e Judiciário a criação de estratégias institucionais, como câmaras técnicas e secretarias específicas para reduzir as distorções sociais, econômicas e políticas ^15^^,^^16^. Estudo de base exploratória das decisões do Tribunal de Justiça de Pernambuco (TJ/PE) ressalta a necessidade de os pleitos judiciais serem subsidiados com evidências científicas consistentes para a tomada de decisão do magistrado ^17^.

Com o objetivo de capacitar os profissionais de saúde que compõem os Núcleos de Apoio Técnico do Poder Judiciário (NatJus), o Conselho Nacional de Justiça (CNJ) e o Ministério da Saúde mantêm cooperação para proporcionar aos magistrados dos Tribunais de Justiça dos Estados e Tribunais Regionais Federais (TRF) subsídios técnicos para a tomada de decisão com base em evidências científicas. Assim, foi criado o Banco Nacional de Pareceres (Sistema e-NatJus) para abrigar os pareceres técnico-científicos e notas técnicas elaboradas pelos NatJus e pelos Núcleos de Avaliação de Tecnologias em Saúde (Nats). Esse sistema é aberto e pode ser acessado pelo *link*https://www.cnj.jus.br/e-natjus/ ^18^.

Considerando a necessidade de promover a discussão sobre o acesso de pacientes aos produtos à base do CBD, e de avaliar as ações judiciais existentes, este estudo tem como objetivo analisar as demandas judiciais movidas contra o Sistema Único de Saúde (SUS) e avaliadas pelos NatJus durante o período de 2019 a 2022.

## Métodos

Trata-se de um estudo transversal, proveniente de pesquisa documental, composto por todas as notas técnicas das ações judiciais demandantes de produtos à base de CBD submetidos ao Ministério da Justiça do Brasil entre dezembro de 2019 e junho de 2022.

A nota técnica é um documento de caráter científico, elaborado pela equipe técnica dos NatJus, que se propõe a responder, de modo preliminar, as questões clínicas sobre os potenciais efeitos de uma tecnologia para uma condição de saúde vivenciada por um indivíduo. Ela é produzida por solicitação de um juiz para auxiliar na tomada de decisão judicial em um caso específico ^18^.

Os dados foram coletados do sistema e-NatJus de forma automatizada, usando técnicas de *web scraping*, desenvolvidas na linguagem de programação Python (https://www.python.org/). De maneira resumida, *web scraping* é um procedimento utilizado para extrair conteúdo da *web* no qual um agente de software, também conhecido como robô *web* (*scraper*), imita a interação entre humanos e servidores *web* em uma navegação convencional na Internet, extraindo e combinando conteúdos de interesse de forma sistemática ^19^.

A extração das variáveis de interesse foi realizada em três etapas. Na primeira, os resultados obtidos da pesquisa feita com o termo “canabidiol” no sistema e-NatJus (https://www.cnj.jus.br/e-natjus/pesquisaPublica.php) foram extraídos com base em algoritmo desenvolvido pelo nosso grupo de pesquisa, os quais incluíram todas as notas técnicas e suas respectivas URL para *download*. A escolha do termo “canabidiol” se deu a partir de alguns testes iniciais para verificar a obtenção de maior número de notas técnicas registradas no sistema e-NatJus.

Na segunda etapa, todas as URL listadas foram consideradas, seus conteúdos foram baixados e salvos em disco local no formato de origem PDF pelo *scraper*. Na última etapa foi criado um segundo algoritmo em Python para extrair e estruturar as variáveis constantes no corpo dos textos das notas técnicas. A lógica de estruturação valeu-se da apresentação consistente das variáveis contidas em cada nota técnica. Ao fim desse processo, as variáveis foram apresentadas em planilha de Excel (https://products.office.com/) para fins de tratamento e análise estatística.

Foram estudadas as características dos pacientes, segundo as categorias das seguintes variáveis: (i) sociodemográficas (sexo, idade e região geográfica de origem); e (ii) diagnósticos médicos por código da Classificação Internacional de Doenças - 10ª revisão (CID-10). As variáveis utilizadas para a caracterização dos pareceres foram: (i) presença ou não de evidências científicas; (ii) registro sanitário do produto na Anvisa; (iii) indicação de uso conforme o registro sanitário; e (iv) avaliação e recomendação pela Comissão Nacional de Incorporação de Tecnologias no SUS (Conitec). Foram utilizadas as variáveis conforme registro feito nas notas técnicas oriundas do sistema e-NatJus.

A análise estatística foi realizada com auxílio do software R, versão 4.0.3 (http://www.r-project.org). As variáveis categóricas foram apresentadas em frequências absolutas e relativas; e as variáveis quantitativas, em valores médios, medianas e desvios padrão.

Uma regressão logística foi realizada para estimar a razão de chances (OR) para investigar a associação entre as variáveis de interesse com o desfecho “parecer favorável para atendimento à demanda judicial”.

O estudo foi aprovado pelo Comitê de Ética em Pesquisa da Fundação de Ensino e Pesquisa em Ciências da Saúde da Secretaria de Estado da Saúde do Distrito Federal (Fepecs-SESDF; parecer nº 5.777.906).

## Resultados

**Figura 1:**
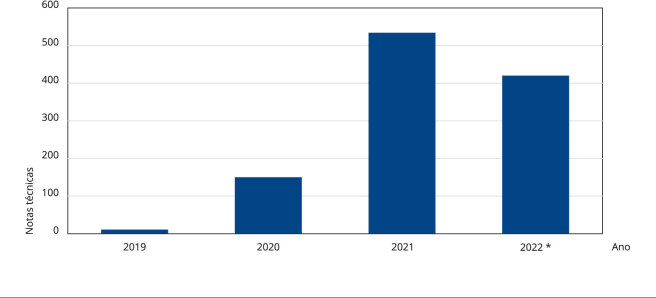
Número de notas técnicas decorrentes de processos judiciais que demandaram produtos à base de canabidiol no Brasil registradas no sistema e-NatJus (n = 1.115).

Foram analisadas todas as 1.115 notas técnicas das ações judiciais demandantes de produtos à base de CBD emitidas pelos NatJus. No ano de 2019, foram inseridas apenas 11 notas técnicas no sistema e-NatJus, valor que foi aumentando progressivamente nos anos seguintes, chegando a 420 até o fim do primeiro semestre de 2022, como mostra a Figura 1.

Fonte: sistema e-NatJus ^18^. * Registros até 30 de junho de 2022.

A Tabela 1 apresenta dados sociodemográficos e clínicos dos pacientes, que eram em sua maioria do sexo masculino (54,7%), tinham idade média de 18,4 anos, com variação de 0 a 90 anos. Grande parte das ações judiciais teve origem na Região Sul do país (38,8%), seguido pela Região Nordeste, com 23,7%. O menor número de ações foi originado na Região Norte (0,9%).

As epilepsias figuraram entre os diagnósticos mais demandados (49,6%), seguidos pelos transtornos globais do desenvolvimento (CID-10 F84) (20,3%). Entre os demais diagnósticos de epilepsia, a epilepsia não especificada (CID-10 G40.9) (7,2%) e outras epilepsias e síndromes epilépticas generalizadas (CID-10 G40.4) (5,4%) foram as mais frequentes (Tabela 1).

**Tabela 1:** Caracterização dos pacientes que demandaram acesso a produtos à base de canabidiol no Brasil no período de 1º de janeiro de 2019 a 30 de junho de 2022 (n = 1.115).

Características	n (%)
Sociodemográficas	
Sexo	
Masculino	610 (54,7)
Feminino	505 (45,3)
Idade em anos (média/DP/mediana)	18,4/19,8/10,0
Origem (região do país)	
Sul	433 (38,8)
Nordeste	264 (23,7)
Sudeste	237 (21,3)
Centro-oeste	171 (15,3)
Norte	10 (0,9)
Clínicas (CID-10)	
Epilepsias	553 (49,6)
Epilepsias não classificadas (G40)	261 (23,4)
Epilepsias classificadas	292 (26,2)
G40.0 - Epilepsia e síndromes epilépticas idiopáticas definidas por sua localização (focal) (parcial) com crises de início focal	26 (2,3)
G40.1 - Epilepsia e síndromes epilépticas sintomáticas definidas por sua localização (focal) (parcial) com crises parciais simples	9 (0,8)
G40.2 - Epilepsia e síndromes epilépticas sintomáticas definidas por sua localização (focal) (parcial) com crises parciais complexas	36 (3,2)
G40.3 - Epilepsia e síndromes epilépticas generalizadas idiopáticas	21 (1,9)
G40.4 - Outras epilepsias e síndromes epilépticas generalizadas	60 (5,4)
G40.5 - Síndromes epilépticas especiais	34 (3,0)
G40.6 - Crise de grande mal, não especificada (com ou sem pequeno mal)	3 (0,3)
G40.8 - Outras epilepsias	23 (2,1)
G40.9 - Epilepsia não especificada	80 (7,2)
F84 - Transtornos globais do desenvolvimento	226 (20,3)
G80 - Paralisia cerebral	44 (3,9)
M79.7 - Fibromialgia	32 (2,9)
R52 - Dor não classificada em outra parte	27 (2,4)
G20 - Doença de Parkinson	24 (2,1)
G30 - Doença de Alzheimer	15 (1,4)
G35 - Esclerose múltipla	9 (0,8)
G50 - Transtornos do nervo trigêmeo	6 (0,5)
Outros *	179 (16,1)

CID-10: Classificação Internacional de Doenças, 10ª revisão; DP: desvio padrão.

Fonte: pareceres e notas técnicas registradas no e-NatJus/Conselho Nacional de Justiça.

* Total de CIDs que representaram menos de 0,3% cada.

**Figura 2:**
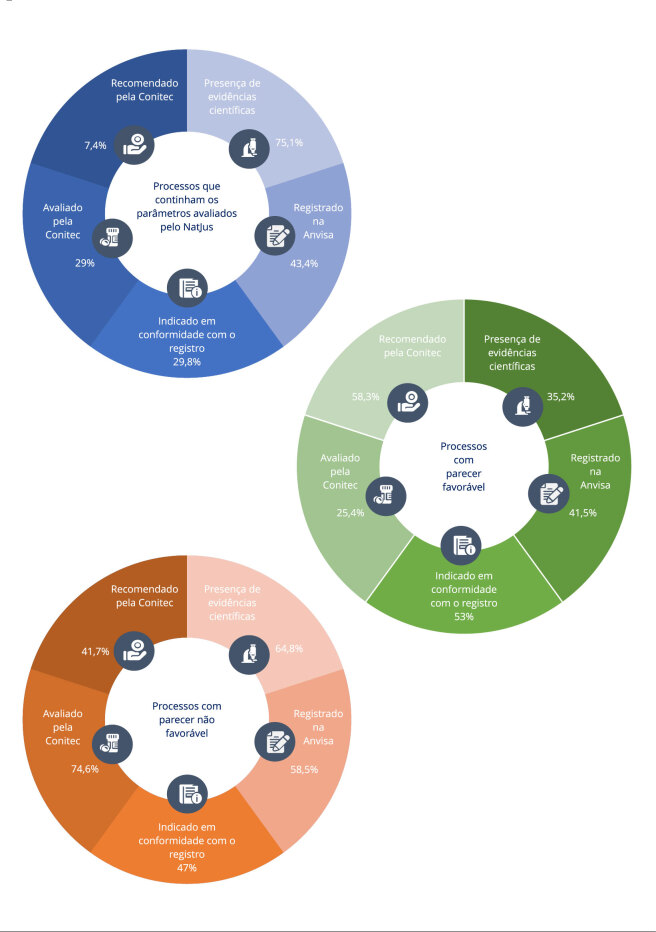
Caracterização dos processos judiciais que demandaram acesso a produtos à base de canabidiol no Brasil no período de 1º de janeiro de 2019 a 30 de junho de 2022 (n = 1.115).

A Figura 2 mostra a estratificação dos pareceres técnicos quanto aos parâmetros avaliados pelos NatJus. Das 1.115 ações judiciais submetidas à avaliação no período, 75,1% eram embasadas em evidências científicas - 35,2% delas tiveram pareceres favoráveis para acesso ao CBD. Dos produtos demandados, 43,4% estavam registrados na Anvisa - 41,5% deles tiveram pareceres favoráveis. Apenas 29,8% das indicações terapêuticas estavam em conformidade com o registro do produto na Anvisa - 53% delas receberam pareceres favoráveis. Das ações judiciais, 29% pleiteavam produtos já avaliados pela Conitec, entretanto, 7,4% deles tinham sido recomendados para incorporação ao SUS. Das ações que demandaram produtos recomendados pela Conitec, 58,3% tiveram pareceres favoráveis ao acesso.

Anvisa: Agência Brasileira de Vigilância Sanitária; Conitec: Comissão Nacional de Incorporação de Tecnologias no SUS. Fonte: sistema e-NatJus ^18^. Nota: a figura foi desenvolvida utilizando ícones do Flaticon (https://www.flaticon.com/).

Dos pareceres das ações que não apresentaram evidências científicas, 28,8% foram ainda assim favoráveis ao acesso. Das solicitações de produtos à base de CBD sem registro na Anvisa, 26,5% foram deferidas. Mesmo pleiteando o uso fora das indicações, 25,3% das ações tiveram parecer favorável quando o produto tinha registro na Anvisa (Tabela 2).

**Tabela 2:** Caracterização dos processos demandantes de acesso ao canabidiol por meio do e-NatJus no período de 1º de janeiro de 2019 a 30 de junho de 2022, Brasil (n = 1.115).

Variáveis	n (%)	Parecer favorável (n = 375)	Parecer não favorável (n = 740)
n (%)	n (%)
Evidências científicas			
Sim	837 (75,1)	295 (35,2)	542 (64,8)
Não	278 (24,9)	80 (28,80)	198 (71,2)
Registro na Anvisa			
Sim	484 (43,4)	201 (41,5)	283 (58,5)
Não	612 (54,9)	162 (26,5)	450 (73,5)
NI	19 (1,7)	12 (63,2)	7 (36,8)
Indicação em conformidade com o registro			
Sim	332 (29,8)	176 (53,0)	156 (47,0)
Não	383 (34,3)	97 (25,3)	286 (74,7)
NI	400 (35,9)	102 (25,5)	298 (74,5)
Avaliação pela Conitec			
Sim	323 (29,0)	82 (25,4)	241 (74,6)
Não	792 (71,0)	293 (37,0)	499 (63,0)
Recomendação pela Conitec			
Sim	24 (7,4)	17 (58,3)	7 (41,7)
Não	299 (92,6)	-	-

Anvisa: Agência Nacional de Vigilância Sanitária; Conitec: Comissão Nacional de Incorporação de Tecnologias no SUS; NI: não informado.

Fonte: pareceres e notas técnicas registradas no e-NatJus/Conselho Nacional de Justiça.

A Tabela 3 relaciona os pareceres para o atendimento da demanda judicial com os parâmetros avaliados e os valores de OR para um parecer favorável, com seus respectivos intervalos de confiança, para uma significância de 5%. Pacientes da Região Nordeste, que tiveram o maior percentual de pareceres favoráveis (51,5%), apresentaram 3,0 vezes mais chances de ter um parecer favorável do que os da Região Centro-oeste. A chance de pacientes com CID-10 G40, que tiveram 43,7% de pareceres favoráveis, terem suas ações deferidas foi 2,3 vezes maior do que indivíduos com CID-10 F84. Os codificados como G40.4, G40.5 e G80 tiveram chances 2,4, 4,8 e 2,0 vezes maiores, respectivamente. Quando há evidência científica para o uso do produto à base de CBD, a chance para um parecer favorável aumenta em 35%. Quando o produto é registrado na Anvisa, a chance de um parecer favorável aumenta em 97%. Ainda, se a indicação terapêutica estiver em conformidade com o registro sanitário, a chance aumenta em 3,3 vezes. Os pacientes que demandam produtos à base de CBD já submetidos à avaliação da Conitec tiveram a chance de um parecer favorável reduzida em 42%.

**Tabela 3:** Razão de chances (OR) para pareceres técnicos favoráveis a processos judiciais que demandaram acesso a produtos à base de canabidiol no Brasil no período de 1º de janeiro de 2019 a 30 de junho de 2022 (n = 1.115).

Variáveis	Parecer favorável	Parecer não favorável	OR	IC95%	Valor de p
	n (%)	n (%)			
Sexo					
Feminino	158 (31,3)	347 (68,7)	1,00	-	-
Masculino	217 (35,6)	393 (64,4)	1,21	0,94-1,56	0,143
Região					
Centro-oeste	43 (25,1)	128 (74,9)	1,00	-	-
Nordeste	136 (51,5)	128 (48,5)	3,01	1,97-4,60	0,000
Norte	4 (40,0)	6 (60,0)	0,95	0,29-3,09	1,000
Sudeste	79 (33,3)	158 (66,7)	1,42	0,91-2,20	0,124
Sul	113 (20,1)	320 (73,9)	1,00	0,67-1,51	1,000
CID-10					
F84	57 (25,2)	169 (74,8)	1,00	-	-
G20	7 (29,2)	17 (70,8)	1,22	0,48-3,09	0,631
G30	2 (13,3)	13 (86,7)	0,46	0,10-2,08	0,372
G35	1 (11,1)	8 (88,9)	0,37	0,05-3,03	0,459
G40	114 (43,7)	147 (56,3)	2,30	1,56-3,39	0,000
G40.0	8 (30,8)	18 (69,2)	1,32	0,54-3,19	0,636
G40.1	1 (11,1)	8 (88,9)	0,37	0,05-3,03	0,459
G40.2	11 (30,6)	25 (60,4)	1,30	0,60-2,82	0,540
G40.3	7 (33.3)	14 (66,7)	1,48	0,57-3,85	0,438
G40.4	27 (45,0)	33 (55,0)	2,43	1,34-4,38	0,004
G40.5	21 (61,8)	13 (38,2)	4,79	2,25-10,18	0,000
G40.6	2 (66,7)	1 (33,3)	5,93	0,53-66,63	0,164
G40.8	9 (39,1)	14 (60,9)	1,91	0,78-4,64	0,213
G40.9	25 (31,2)	55 (68,8)	1,35	0,77-2,36	0,306
G50	2 (33,3)	4 (66,7)	1,48	0,26-8,31	0,646
G80	18 (40,9)	26 (59,1)	2,05	1,05-4,02	0,043
M79.7	4 (12,5)	28 (87,5)	0,41	0,14-1,21	0,124
R52	9 (33,3)	18 (66,7)	1,48	0,63-3,48	0,361
Outros *	47 (26,3)	132 (73,7)	1,14	0,73-1,79	0,566
Evidência científica					
Não	80 (28,8)	198 (71,2)	1,00	-	-
Sim	295 (35,2)	542 (64,8)	1,35	1,00-1,81	0,048
Registro na Anvisa					
Não	162 (26,5)	450 (73,5)	1,00	-	-
Sim	201 (41,5)	283 (58,5)	1,97	1,53-2,55	0,000
Indicação conforme registro					
Não	97 (25,3)	286 (74,7)	1,00	-	-
Sim	176 (53,0)	156 (47,0)	3,33	2,43-4,56	0,000
Avaliação pela Conitec					
Não	293 (37,0)	499 (63,0)	1,00	-	-
Sim	82 (25,4)	241 (74,6)	0,58	0,43-0,77	0,000

Anvisa: Agência Nacional de Vigilância Sanitária; CID-10: Classificação Internacional de Doenças, 10ª revisão; Conitec: Comissão Nacional de Incorporação de Tecnologias no SUS; IC95%: intervalo de 95% de confiança.

Fonte: pareceres e notas técnicas registradas no e-NatJus/Conselho Nacional de Justiça.

* Total de CIDs que representaram menos de 0,3% cada.

## Discussão

Os dados obtidos no sistema e-NatJus não permitiram traçar um perfil mais preciso dos pacientes que demandaram o CBD, caracterizando, assim, uma limitação deste estudo. Em pesquisa recente que avaliou ações judiciais de solicitação de medicamentos, os autores relatam que, mesmo após mais de duas décadas da existência do fenômeno de judicialização no Brasil, ainda há dificuldade para traçar um perfil nacional das demandas e dos demandantes. Os autores avaliam, ainda, que dados de estudos locais, realizados em sua maioria nas regiões Sul e Sudeste do país, não permitem extrapolações, principalmente para as realidades socioeconômicas profundamente distintas, como as observadas nas regiões Norte e Nordeste ^20^.

A maior parte das ações judiciais teve origem na Região Sul do país; e a menor, na Região Norte. Essa relação pode ter sido influenciada pela diferença socioeconômica das populações dessas duas regiões e pelos níveis de acesso à informação e aos serviços de saúde. Ao avaliar os fatores associados aos serviços de saúde, estudo recente concluiu que a população da Região Norte apresenta maior precarização no acesso e que o nível de acesso da Região Sul se aproxima ao da Região Sudeste ^21^. Pesquisas têm demonstrado que a litigação para acesso a medicamentos e produtos para a saúde é determinada por aspectos sociais e políticos e que pode agravar a iniquidade no sistema de saúde nos casos em que ações judiciais são movidas por pacientes oriundos do setor privado ^15^^,^^16^^,^^22^.

As condições de epilepsias figuraram entre os diagnósticos que mais demandaram acesso ao produto, seguidas pelos transtornos globais do desenvolvimento (CID-10 F84). Entre os diagnósticos de epilepsias classificadas, a epilepsia não especificada (CID-10 G40.9), outras epilepsias e síndromes epilépticas generalizadas (CID-10 G40.4) foram as mais frequentes. A prevalência desses diagnósticos pode ser explicada pelo fato de vários estudos terem evidenciado eficácia e segurança do uso do CBD no tratamento dessa doença e do transtorno do espectro autista, apesar de ainda serem necessários ensaios clínicos randomizados, cegos e controlados para esclarecer os efeitos do CBD ^4^^,^^6^^,^^7^^,^^8^^,^^9^^,^^10^^,^^23^^,^^24^^,^^25^^,^^26^^,^^27^^,^^28^.

A maioria das solicitações apresentou evidência científica para o tratamento indicado, entretanto, somente 1/3 destas tiveram pareceres favoráveis para o acesso ao CBD. Dos pareceres sem evidências científicas, 28,8% foram mesmo assim favoráveis ao acesso por ordem judicial. Percebe-se que a falta de evidência científica não foi fator preponderante para pareceres favoráveis ao acesso, podendo a prescrição médica ter prevalecido como documento suficiente para atendimento à demanda judicial. Apesar da busca incessante por evidência científica que embase a prescrição do CBD e sua disponibilização pelo SUS, sua eficácia comprovada permanece restrita às condições de epilepsias pediátricas resistentes a tratamentos com medicamentos convencionais ^23^^,^^24^.

Os achados evidenciam uma deficiência de rigor científico nas discussões para as decisões judiciais para acesso ao CBD por gravitarem em torno de pretensos direitos não reconhecidos por tratamentos e produtos sem registros na Anvisa. Apesar da *Lei nº 6.360/1976*^29^ exigir que medicamentos e produtos para a saúde devam passar por testes que comprovem cientificamente a eficácia e a segurança, o Supremo Tribunal Federal (STF) decidiu no julgamento do Recurso Extraordinário 657.718/MG ^30^, em 22 de maio de 2019, que a concessão judicial sem registro sanitário pode, excepcionalmente, ocorrer em caso de demora irrazoável da agência em apreciar o pedido de registro (prazos superiores aos previstos na *Lei nº 13.411/2016*^31^), quando preenchidos os seguintes requisitos: a existência de pedido de registro no Brasil, salvo no caso drogas órfãs para doenças raras; a existência de registro do produto em renomadas agências de reguladores estrangeiras; e a inexistência de substituto terapêutico com registro no país. Essa decisão do STF, com a intenção inicial de frear a proliferação de deliberações judiciais que condenam o Estado a fornecer medicamentos e produtos sem registro e de alto custo, manteve aberta a possibilidade de concessão por via judicial, mesmo na ausência de registro na Anvisa ^29^^,^^30^^,^^31^.

O estudo demonstrou a emissão de pareceres favoráveis para o acesso ao CBD por via judicial, mesmo quando a prescrição estava fora das indicações terapêuticas conforme o registro do produto na Anvisa. A prescrição e a concessão judicial para uso *off-label* (fora das indicações dos produtos à base de CBD registrados na agência) podem estimular a divulgação do CBD como uma panaceia para uma ampla gama de problemas de saúde e tentativa de comercialização para outros fins, como dietético e de bem-estar ^32^.

Das ações judiciais, 29% pleiteavam produtos já avaliados pela Conitec, entretanto, apenas 7,4% deles tinham sido recomendados para incorporação ao SUS. Das ações que demandaram produtos recomendados pela Conitec, 58,3% tiveram pareceres favoráveis ao acesso.

A incorporação ao SUS de produtos para a saúde deve ser baseada em evidências científicas de eficácia e segurança, além de avaliação econômica e de impacto orçamentário, produzidas por estudos de avaliação de tecnologia em saúde (ATS). Após análises desses estudos, cabe à Conitec tomar a decisão sobre a incorporação. Percebe-se nesse contexto a importância do alinhamento das tomadas de decisões entre o Poder Judiciário e a Conitec, no sentido de garantir a disponibilização à população de medicamentos e produtos como o CBD, demandados judicialmente ^33^^,^^34^.

O estudo demonstrou que a chance de um parecer ter sido deferido foi 3,0 vezes maior em ações judiciais da Região Nordeste do que da Região Centro-oeste; 2,3 vezes maior para pacientes com diagnóstico de epilepsias não classificadas (CID-10 G40) do que para aqueles com diagnóstico de transtornos globais do desenvolvimento (CID-10 F84); 2,4, 4,8 e 2,0 vezes maiores que aqueles com diagnóstico de outras epilepsias e síndromes epilépticas generalizadas (CID-10 G40.4), síndromes epilépticas especiais (CID-10 G40.5) e paralisia cerebral (CID-10 G80); 35% maior quando havia evidência científica; 97% maior quando o produto era registrado na Anvisa; e 3,3 vezes maior se a indicação terapêutica estivesse em conformidade com o registro. Por outro lado, produtos à base de CBD já submetidos à avaliação da Conitec tiveram a chance de um parecer favorável reduzida em 42%.

A prescrição de produtos à base de CBD, mesmo na ausência de evidências científicas robustas, pode estar refletindo decisões clínicas que consideram prioritariamente os anseios do paciente e a concepção do médico de estar proporcionando um “benefício” para o indivíduo. Tal fato pode também refletir negativamente sobre a abordagem social do uso do CBD, aprofundando vieses ideológicos e discursos de cunho preconceituoso. Estimular a realização de estudos de considerável robustez, que avaliem os promissores efeitos benéficos do CBD em diferentes condições clínicas, seria uma importante estratégia para oferecer maiores garantias de resultados positivos durante seu uso.

As maiores chances para se terem pareceres favoráveis para demandas com diagnóstico de epilepsias e com indicação terapêutica, de acordo com o registro dos produtos à base de CBD na Anvisa, demonstram que os pareceres e as decisões judiciais podem estar de acordo com os estudos que evidenciam o uso de CBD para o tratamento de epilepsias de difícil tratamento com as terapias convencionais ^3^^,^^4^^,^^5^^,^^6^^,^^7^^,^^8^^,^^9^.

A impossibilidade de captura de dados nas notas técnicas emitidas pelos NatJus, como o tipo de advocacia, se pública ou privada, e a qual estabelecimento de saúde o profissional que emitiu o relatório médico estaria vinculado, pode ter sido fator de limitação do estudo. Outra importante limitação foi a quase inexistência de pesquisas abordando a judicialização de produtos à base de CBD, para efeito de comparação.

Os achados deste estudo podem contribuir para o aprimoramento técnico dos diferentes atores com poder decisório envolvidos no processo de judicialização (profissionais de saúde, juristas e gestores públicos), garantindo, assim, o acesso de pacientes demandantes de produtos à base de CBD com eficácia e segurança evidenciadas para tratamento de suas condições clínicas. O estudo pode também possibilitar uma reflexão, sem viés ideológico, sobre a possibilidade de padronização no SUS dos produtos à base de CBD, inicialmente para epilepsias de difícil tratamento.

## Conclusão

Conclui-se que os pareceres técnicos que deram suporte aos magistrados para as decisões judiciais das demandas de pacientes por produtos à base de CBD no Brasil estavam, em sua maioria, em conformidade com evidências científicas, denotando a importância dos NatJus na qualificação do acesso a produtos medicinais no país por essa via. No entanto, a falta de evidência científica e o uso fora da indicação terapêutica, quando registrados na Anvisa, não foram fatores preponderantes para a emissão de pareceres técnicos favoráveis ao acesso de produtos à base de CBD.
